# Wheat Breeding through Genetic and Physical Mapping

**DOI:** 10.3390/ijms21228739

**Published:** 2020-11-19

**Authors:** Agata Gadaleta

**Affiliations:** Department of Agricultural and Environmental Science, University of Bari Aldo Moro, 70121 Bari, Italy; agata.gadaleta@uniba.it

The Special Issue of “Wheat breeding through genetic and physical mapping” aimed to collect recent advances in research on the genetic and physical mapping of quantitative trait loci (QTLs), candidate genes and regulatory sequences involved in the control of wheat’s important agronomic traits, such as grain yield and quality, biotic and abiotic stress resistance. In a very short time, this Special Issue attracted attention from the scientific research community, including a total of 10 published papers, of which two were review papers focusing on two main subjects for wheat breeding: yield and quality. Ariagada and colleagues [1] summarized all the current studies in durum wheat, identifying quantitative trait loci (QTLs) and candidate genes involved in the main traits linked to grain yield: kernel weight, number of kernels per spike and number of spikes per unit area. 

Durum wheat provides important cultural and commercial benefits in the Mediterranean regions, but the crop may be seriously affected by climate change, which can produce yield losses of up to 50% [2]. In particular, authors highlight the recent results obtained by the scientific community on the identification of the genetic regions that regulate grain yield and QTLs for kernel weight, number of kernels per spike and number of spikes per unit area that are the most important components affecting grain yield. Besides a section with candidate genes recognized as being associated to the traits, genes involved in the regulation of cell proliferation and cell elongation, flowering control, spike elongations and genes for transcription factors and signaling proteins, participating in the regulation of carbon and nitrogen metabolism, kernel and plant development and biotic and abiotic stress responses were reported [1]. 

The second review paper by Marcotuli et al. [3] summarizes the recent knowledge of the genetic control of fiber content in wheat with particular attention on synthesis and accumulation of two specific fiber components, arabinoxylan and (1,3;1,4)-β-glucan. The characterization of specific plant materials and the release of the durum wheat genome sequences, together with the development of more accurate classes of DNA-based markers and consensus maps, have allowed the identification of important genes involved in the control of (1,3;1,4)-β-glucan and arabinoxylan biosynthesis. Dietary fiber comprises cell wall polymers and includes insoluble and soluble components such as arabinoxylans and (1,3;1,4)-β-glucans fiber [4]. These components have been demonstrated to provide immunomodulatory and cholesterol lowering activity, fecal bulking effects, enhanced absorption of certain minerals, prebiotic effects and, through these effects, reduce the risk of type II diabetes, cardiovascular disease and colorectal cancer. Advanced research on this topic can help breeding programs to generate advanced lines with high fiber content.

Many QTL regions and candidate genes have been described in the review as being involved in the control of (1,3;1,4)-β-glucan and arabinoxylan [5,6]. Additionally, the isolation and characterization of the *CslF6* and *CslH* durum gene sequences have been reported together with the expression pattern in durum endosperm at different developmental stages, increasing the speed of the genetic gains [7].

The remaining eight research papers of this Special Issue are focused on studies of principal wheat disease such as crown rot, stem rust, Septoria leaf blotch and studies on yield and quality traits in different genetic materials with a basic study on the distribution and correspondence of genome-scale homologous genes in wheat. Zhou et al. [8] presented an effective way to decipher the correspondence of homologous genes and chromosome rearrangement that occurs during polyploid *Triticum* species evolution or the domestication process. The main results included the identification of three regions of exceptional density detected in 1:1:1 homologous genes, a peak on the tail of chromosome 4A and the desert regions at the start of chromosomes 7A and 7D. Chromosomes rearrange, for example, the translocation between the tail segments of chromosome 4A and 5A and the inversion of the segment of original 5A and 7B into the tail of 4A [8].

A study of the incidence of crown rot (CR) on yield in field experiments as well as root traits under controlled conditions was reported by Alahmad et al. [9]. Researchers identified quantitative trait loci (QTLs) through a GWAS (Genome Wide Association Study) using DArTseq markers. A QTL for CR tolerance and stay-green was detected on chromosome 6B (qCR-6B) with a major QTL for root angle on 6A (qSRA-6A) detected using yield datasets from six rainfed environments, including two environments with high CR disease pressure. Results of this study highlight the value of combining above- and belowground physiological traits to enhance wheat yield potential [9].

Stem rust caused by *Puccinia graminis* f. sp. *tritici* Eriks was studied by Leonova and colleagues [10] through a genome-wide association study with SNP markers in a collection of different genotypes. The collection consisting of Russian varieties of spring wheat was evaluated for resistance to the native population of stem rust specific to the West Siberian region of Russia. GWAS detected 35 significant marker-trait associations (MTAs) with SNPs located on chromosomes 1A, 2A, 2B, 3B, 5A, 5B, 6A, 7A and 7B. The most significant associations were found on chromosomes 7A and 6A confirming known resistance genes *Sr25* and *Sr6Ai* originated from *Thinopyrum* ssp. 

To the best of the authors’ knowledge, this is the first report describing an analysis of the genetic factors conferring resistance of Russian spring wheat varieties to stem rust [10].

Poll et al. [11] present an interesting study on the relationship between Septoria resistance and plant nitrogen availability. Septoria leaf blotch caused by *Zymoseptoria tritici* is one of the major causes of yield loss in wheat worldwide and in particular in Europe. Little resistance has been presented in commercial wheat varieties for this pathogen even if a link between reduced nitrogen availability and increased Septoria tolerance has been observed. The authors confirmed that a link between nitrogen and Septoria is only present during the necrotrophic phase of fungus infection through a quantitative real-time PCR for *WRKYs*, a superfamily of plant-specific transcription factors, that were found to be differentially expressed in response to both reduced nitrogen and Septoria. *WRKY39* was downregulated in response to the necrotrophic stage of Septoria, whilst changes in the expression of *WRKY68a* during the late biotrophic phase were dependent on the concentration of nitrogen under which wheat is grown [11]. In the manuscript from Karim et al. [12], data were instead presented on a comparison of the adaptive processes to N deficiency with different Al-tolerant wheat cultivars; two cultivars were chosen—Atlas 66 and Scout 66—to comprehensively analyze the physiological responses to N deficiency, coupled with label-free mass spectrometry-based proteomics analysis [12].

Results showed the identification of differentially expressed proteins involved in cellular N compound metabolic process, photosynthesis, etc. The manuscript suggested the need for a better understanding of genotype-dependent plant responses under N deficiency which could help breeders to develop N efficient cultivars. 

Shokat et al. [13] present an example of the use of pre-breeding lines to study terminal drought stress. Drought stress was executed at flowering time skipping the irrigations at pre- and post-anthesis stage. Results revealed that drought significantly reduced grain yield and its component traits, spike length, number of grains spikes−1 and thousand kernel weight, while only kernel abortion was increased. Phenotypic data were compared with genotypic data, and a genome-wide association study allowed the identification of chromosome regions associated to the target traits on 4A (HB10.7), 2D (HB6.10), 3B (HB8.12), 6B (HB17.1) and 3A (HB7.11) chromosomes [13]. 

The same traits were studied by Liu et al. [14] with a different approach. The authors concentrated their efforts on a yield component, thousand-grain weight, a very important yield trait for crops. A classical QTL analysis of Thousand-grain weight (TGW) was applied in a doubled haploid population obtained from a cross between the bread wheat cultivar “Superb” and the breeding line “M321” using the wheat 55-K SNP genotyping assay. This allowed the detection and mapping of 9 QTLs on chromosomes 1A, 2D, 4B, 4D, 5A, 5D, 6A and 6D expressed in six experimental environments. [14].

The last manuscript, here described, is from Parada et al. [15] and reported a deep study on yellow color that with protein quantity and composition of seed storage proteins are the most important and studied quality traits in wheat [16]. A candidate gene approach was used to study the yellow color of semolina. New haplotype variants and/or allele combinations of phytoene synthase 1 (*Psy1*) and lipoxygenase 1 (*Lpx-1*) genes involved in the synthesis and catalysis of carotenoid pigments were detected, in addition to a QTL analysis that confirmed that *Psy1* plays a major role during grain development, contributing to semolina yellowness, and *Lpx-1* appears to be more predominant at post-harvest stages and during pasta making.

In conclusion, in the present Special Issue, several important tools affecting wheat genetics reported in the data may help breeders to design improved durum varieties that mitigate production losses and improve quality. I wish to thank all the authors for their significant contribution to the collection of these articles as well as the support of the International Journal of Molecular Science.
